# Current Advances in the Diagnosis and Treatment of Major Myeloproliferative Neoplasms

**DOI:** 10.3390/cancers17111834

**Published:** 2025-05-30

**Authors:** Le Wang, Julie Li, Leah Arbitman, Hailing Zhang, Haipeng Shao, Michael Martin, Lynn Moscinski, Jinming Song

**Affiliations:** 1 Department of Hematology and Oncology, Guthrie Clinic Cancer Center, Robert Packer Hospital, Sayre, PA 18840, USA; larbitm1@binhangton.edu; 2 Department of Hematopathology and Lab Medicines, H. Lee Moffitt Cancer Center & Research Institute, Tampa, FL 33612, USA; julie.li@moffitt.org (J.L.); hailing.zhang@moffitt.org (H.Z.); haipeng.shao@moffitt.org (H.S.); lynn.moscinski@moffitt.org (L.M.); 3 University of South Florida Morsani College of Medicine, Tampa, FL 33612, USA; michaelseanmartin@usf.edu

**Keywords:** myeloproliferative neoplasms (MPNs), chronic myeloid leukemia (CML), polycythemia vera (PV), essential thrombocythemia (ET), primary myelofibrosis (PMF), fusion gene between the breakpoint cluster region-Abelson murine leukemia viral oncogene homolog 1 (BCR::ABL1), Janus kinase 2 (JAK2), cytogenetics response (CyR), molecular response (MR), minimal residual disease (MRD

## Abstract

Myeloproliferative neoplasms (MPNs) are a group of rare blood cancers characterized by the excessive production of blood cells in the bone marrow. These disorders arise from acquired genetic driver mutations, with or without underlying genetic predispositions, resulting in the uncontrolled production of red blood cells, white blood cells, or platelets. The excessive cell production and abnormal signaling from driver mutations cause chronic inflammation and a higher risk of blood clots and vascular complications. The primary goals of MPN treatment are to induce remission, improve quality of life and survival, as well as to reduce the risk of complications such as thrombosis, vascular events, and leukemic transformation. This review provides a comprehensive update on the diagnosis and therapeutic advancements in major MPN subtypes, including chronic myeloid leukemia, polycythemia vera, essential thrombocythemia, and primary myelofibrosis. It examines these complex diseases from a molecular and evolutionary perspective, highlighting key clinical trials’ long-term follow-up and therapies targeting driver mutations that have transformed treatment strategies. Additionally, several important advancements in addressing challenges such as anemia in myelofibrosis, along with promising emerging therapies, are also discussed.

## Simple Summary

Myeloproliferative neoplasms (MPNs) are rare blood cancers caused by genetic mutations that lead to the excessive production of blood cells in the bone marrow. This uncontrolled proliferation increases the risk of chronic inflammation, blood clots, and vascular complications. Treatment aims to induce remission, improve survival and quality of life, and prevent severe complications like thrombosis and leukemic transformation. This review provides an overview of major MPN subtypes, including chronic myeloid leukemia, polycythemia vera, essential thrombocythemia, and primary myelofibrosis. It examines molecular mechanisms underlying these diseases, key clinical trials’ long-term follow-up, and recent FDA-approved therapies that target driver mutations of the diseases, advancing treatment options significantly. This article also highlights new strategies for managing anemia in myelofibrosis and explores emerging therapies that show promise for improving patient outcomes.

## 1. Introduction

Myeloproliferative neoplasms (MPNs) are a group of rare clonal disorders originating from myeloid hematopoietic stem cells (HSCs). They are broadly classified into two categories: *BCR::ABL1*-positive chronic myeloid leukemia (CML) and *BCR::ABL1*-negative MPNs. CML is characterized by the excessive production of neutrophils and their precursors, while *BCR::ABL1*-negative MPNs are primarily driven by mutations in *JAK2*, *CALR*, or *MPL*. These include polycythemia vera (PV), essential thrombocythemia (ET), primary myelofibrosis (PMF), chronic neutrophilic leukemia (CNL), chronic eosinophilic leukemia, and MPN-NOS/unclassifiable [1,2]. Up to 10% of ET, PMF, or PV may be triple-negative.

Each subtype of Ph-negative MPNs exhibits distinct hematologic and molecular characteristics. Advances in next-generation sequencing (NGS) and polymerase chain reaction (PCR) have significantly enhanced our understanding of the mutational landscape of MPNs by identifying genome-wide genetic variants and measuring variant allele frequency (VAF). The 5th edition of World Health Organization of Tumors of the Hematopoietic and Lymphoid Tissues (WHO-HEM5) and the International Consensus Classification (2022 ICC) places greater emphasis on genetic features compared to the 2016 classification [1,2]. The new classification and diagnosis guidelines underscore the importance of integrating genetic, morphological, and clinical data for accurate diagnosis and effective planning for the treatment of MPNs. This review provides a focused update on the diagnosis and the therapeutic advances in CML, PV, ET, and PMF.

## 2. Clinical Features, Diagnosis, and Classification

### 2.1. Chronic Myeloid Leukemia (CML)

CML is the prototypical example of Philadelphia chromosome-positive MPNs (Ph+ MPNs). It is defined by a reciprocal translocation between the Abelson oncogene (*ABL1*) on chromosome 9 and the breakpoint cluster region (*BCR*) gene on chromosome 22, creating the well-known Philadelphia chromosome (Ph) [3,4,5]. The resulting *BCR::ABL1* fusion protein is the primary driver of CML by acting as a constitutively active tyrosine kinase. It stimulates myeloid proliferation through several downstream signaling pathways, including *RAS*, *RAF*, *JUN* kinase, *MYC*, and *STAT* [6]. The most common breakpoint in the *ABL1* gene occurs in the intron before exon 2 (a2), with rare breakpoints downstream of exon 2 (a3). The *BCR::ABL1* fusion gene has three transcript variants due to different breakpoints in the *BCR* gene: the major breakpoint cluster region (M-bcr), minor breakpoint cluster region (m-bcr), and micro breakpoint cluster region (µ-bcr). M-bcr breakpoints occur downstream of exon 13 (e13) or exon 14 (e14) of the *BCR* gene, producing the p210 fusion protein, which is found in 97–99% of CML cases and in 40% of adult and 10% of pediatric B-lymphoblastic leukemia/lymphoma (B-ALL) patients. The m-bcr breakpoints occur after exon 1 (e1) of the *BCR* gene, resulting in the smaller p190 fusion protein. CML with p190 (m-bcr) can coexist with p210 in some CML patients, with a prevalence of 5–7% in some studies, and resembles chronic myelomonocytic leukemia (CMML) with increased monocytes. p190 is also more commonly associated with Ph-positive B-ALL (60% of adult and 90% of pediatric cases). The µ-bcr breakpoints occur beyond exon 19 (e19) of the *BCR* gene, leading to the larger p230 oncoprotein, which is rare and associated with neutrophilic CML and characterized by predominant neutrophilic maturation and/or thrombocytosis [7]. The diagnosis of CML requires clinical–pathological correlation and cannot be made solely based on the presence of *BCR::ABL1*, as this fusion gene has also been detected in healthy individuals [8,9].

CML is relatively uncommon, accounting for approximately 0.5% of all new cancer cases in the United States. According to The Surveillance, Epidemiology, and End Results (SEER) data, there were around 9280 new cases of CML diagnosed and approximately 1280 deaths from the disease in 2024 [10]. The 5-year relative survival rate for CML was 70% from 2014 to 2020 [10]. Clinically, CML can be classified into three phases: the chronic phase (CP), accelerated phase (AP), and blast phase (BP). The primary manifestation of CML is leukocytosis, which may present with or without symptoms. Most patients with CML (over 90%) are diagnosed during the chronic phase. Common signs and symptoms include constitutional symptoms such as fever, night sweats, weight loss, fatigue, early satiety, and left upper quadrant pain due to splenomegaly. Occasionally, patients with CML may also experience bleeding or thrombosis (associated with thrombocytosis), or leukostatic symptoms (e.g., dyspnea, drowsiness, loss of coordination, confusion) due to leukemic cells clogging the pulmonary or cerebral vessels. In the AP phase, there is a marked rise in white blood cell (WBC) count, an increase in blasts in both blood and bone marrow (10–19%), and a decrease in platelets. In the BP phase, the patient effectively progresses to acute leukemia, characterized by severe symptoms and blast counts exceeding 20% in either blood or bone marrow.

The diagnostic criteria for CP-CML and BP-CML CML have remained consistent over time. CP-CML is defined by the presence of the *BCR::ABL1* fusion gene, while BP-CML is diagnosed when blasts in peripheral blood (PB) or bone marrow (BM) exceed ≥20%, or when myeloid sarcoma or lymphoblast infiltration is present. The latter requires >5% lymphoblasts per International Consensus Classification (ICC) criteria, with no specified cut-off in the World Health Organization (WHO) 5th edition. However, the diagnostic framework for AP-CML has undergone significant updates in both the 2022 ICC and the WHO 5th edition. While the ICC continues to recognize three phases of CML (CP, AP, and BP), it has simplified the criteria for diagnosing AP-CML to three primary diagnostic parameters. In contrast, the WHO 5th edition has removed the AP designation, acknowledging only CP-CML and BP-CML. This change reflects the view that the AP-CML designation is less clinically important in the era of tyrosine kinase inhibitor (TKI) therapy and may be better referred to as “high-risk CP”.

The prognosis for CML is often determined by using several scoring systems that help predict outcomes and guide treatment decisions. The three most commonly used prognostic scoring systems are the Sokal Score, Hasford Score, and European Treatment and Outcome Study (EUTOS)/Long-Term Survival (ELTS) Score. These systems take into account the factors such as age, spleen size, platelet count, and the percentage of myeloblasts in the peripheral blood or bone marrow. However, the EUTOS/ELTS, being a newer scoring system, places greater emphasis on the percentage of basophils in the blood [11]. In the 2022 updates, the ELTS score, along with TKI resistance, additional cytogenetic abnormalities, and *ABL1* kinase mutations, are now considered when diagnosing high-risk CP- or AP-CML (Table 1). The ELTS score can be computed using an online tool (EUTOS Score, https://www.mdcalc.com/calc/3906/eutos-score-chronic-myelogenous-leukemia-cml, accessed on 25 March 2025).

### 2.2. Ph-Negative MPN: PV, ET, PMF

PV, ET, and PMF are the three major classic MPNs, constituting approximately 50–60%, 20–25%, and 15–20% of all MPN cases, respectively. These disorders are associated with specific mutations in the *JAK2*, *CALR*, and *MPL* genes, known as driver mutations. PV is characterized by erythrocytosis, often accompanied by leukocytosis or thrombocytosis, and carries an increased risk of thrombotic and hemorrhagic events. ET is marked by persistently elevated platelet counts and increased large, mature, and hyperlobated megakaryocytes in the bone marrow, also associated with thrombotic or hemorrhagic complications. PMF involves abnormal megakaryocyte and granulocyte proliferation, progressing from prefibrotic/early PMF (hypercellular marrow with minimal fibrosis) to fibrotic stage PMF (advanced fibrosis with leukoerythroblastosis, anemia, splenomegaly, and/or hepatomegaly). All three MPNs can progress to accelerated-phase disease (10–19% blasts in peripheral blood or bone marrow) or blast-phase disease (≥20% blasts in peripheral blood or bone marrow), indicative of leukemic transformation.

#### 2.2.1. Polycythemia Vera (PV)

PV, first described by French physician Louis Henri Vaquez in 1892, is primarily characterized by erythrocytosis due to the excessive production of blood cells in the bone marrow. To a lesser extent, PV also presents leukocytosis and thrombocytosis. Important clinical features include constitutional symptoms, pruritus, splenomegaly, microcirculatory disturbances, and an increased risk of thromboembolic events. Neurological and thromboembolic complications, such as ischemic stroke, can occur in 15 to 33% of PV patients and are common causes of morbidity and mortality in these individuals. The early identification of neurological manifestations through neuroimaging, combined with a clinical diagnosis of PV, can guide clinicians towards timely diagnosis and can help limit morbidity and mortality [12]. The risk of arterial and venous thrombosis, and the transformation to myelofibrosis (post-PV MF) and acute leukemia, is directly related to *JAK2* V617V mutation burden [13,14]. The 20-year incidence rates for thrombosis, post-PV MF, or leukemia are 26%, 16%, and 4%, respectively [13].

The major diagnostic criteria for PV, as summarized in Table 2, include elevated hemoglobin/hematocrit levels of 16.5g/dL/49% in men or 16g/dL/48% in women, and the presence of the *JAK2* gene mutation. The only minor criterion is a subnormal erythropoietin level, which helps differentiate PV from secondary erythrocytosis caused by non-neoplastic factors such as smoking, sleep apnea, and testosterone use. Poor outcomes are associated with older age, leukocytosis, abnormal karyotype, and the presence of high-risk mutations in the *SRSF2*, *IDH2*, *RUNX1*, and *U2AF1* genes [15]. Additionally, higher absolute neutrophil counts and the *JAK2* V617F allele burden are linked to an increased risk of venous thrombosis [15].

#### 2.2.2. Essential Thrombocythemia (ET)

ET, first described by Epstein and Goedel in 1934, is characterized by a significantly elevated platelet count in the blood and hyperplasia of megakaryocytes in the bone marrow. This condition increases the risk of both venous thrombosis and spontaneous hemorrhage [16]. The annual incidence of ET is comparable to that of PV, with approximately 1.5 to 2.0 cases per 100,000 people. The median age of onset is between 60 and 70 years, with elderly patients at a particularly increased risk of bleeding [17]. Although the overall clinical course of ET is generally indolent, it can be complicated by microcirculatory symptoms such as headache, lightheadedness, and acral paresthesia, as well as splenomegaly. Less frequently, ET may progress to myelofibrosis (MF) and acute myeloid leukemia (AML) [18].

ET is diagnosed by persistent thrombocytosis greater than 450 × 10^9^/L, in the absence of reactive thrombocytosis or alternative myeloid neoplasm, and evidence of hyperplasia of megakaryocytes in the bone marrow. A large observational study of one-thousand ET patients showed that nearly 90% had mutations in one of three key driver genes, which are mutually exclusive: *JAK2* (62%), *CALR* (27%), and *MPL* (3%), with 8% being triple-negative [19]. Co-mutations are found in 50% of ET patients, with the most common being *TET2* (9–11%), *ASXL1* (7–20%), *DNMT3A* (7%), and *SF3B1* (5%). A small number of patients (<10%) present with abnormal karyotypes, such as +9, 20q−, and 13q− [18]. Mutations in the *JAK2*, *MPL*, or *CALR* genes are associated with an increased risk of progression to MF. Spliceosome mutations, such as those in *SF3B1*, *SRSF2*, *U2AF1*, and *ZRSR2*, are commonly found in myeloid neoplasms like myelodysplastic syndromes (MDSs) and other MPNs [20]. These mutations can lead to defective pre-mRNA splicing, contributing to leukemogenesis [21]. Specifically, *SRSF2* mutations have been identified as playing a significant role in the pathogenesis of chronic myelomonocytic leukemia (CMML) and MPNs [22]. Spliceosome mutations are linked to decreased overall survival (OS) and MF-free survival, while *TP53* mutations carry an increased risk for leukemic transformation [23,24]. *JAK2* V617F mutations are also associated with a higher risk of thrombosis and leukemic transformation, particularly when combined with extreme thrombocytosis and/or abnormal karyotypes [18].

Patients with ET typically have a normal life expectancy when properly monitored and treated, according to the Leukemia & Lymphoma Society. According to the Triple A survival risk model (age, absolute neutrophil count, and absolute lymphocyte count), ET can be categorized into four risk groups, with median survivals of 8, 14, 21, and 47 years, respectively [25]. The very low-risk group includes patients aged 60 years or younger with no history of thrombosis and wild type *JAK2*. The low-risk group comprises patients with the same characteristics but with a *JAK2* mutation. The intermediate-risk group includes patients with the same characteristics as the low-risk group but aged over 60 years. The high-risk group consists of patients with a history of thrombosis or those aged over 60 years with a *JAK2* mutation.

#### 2.2.3. Primary Myelofibrosis (PMF)

PMF is characterized by the presence of reticulin/collagen fibrosis in the bone marrow, anemia, extramedullary hematopoiesis (EMH), constitutional symptoms, cachexia, and an increased risk of leukemic transformation [26]. Splenomegaly is a key clinical manifestation of PMF, along with recurrent anemia and progressive cytopenia. Other symptoms include easy bleeding, bruising, frequent infections, and bone pain. Up to 20% of PMF patients may transform into acute myeloid leukemia. In very rare cases, the extramedullary blastic transformation of PMF can occur in the form of disseminated myeloid sarcoma [27].

*JAK2*, *CALR*, and *MPL* are pivotal driver mutations in PMF. Co-mutations in genes such as *ASXL1*, *DNMT3A*, *SRSF2*, *EZH2*, and *IDH1/2* are increasingly recognized for their roles in PMF [22,26,28,29,30,31,32,33,34,35]. Mutations in *SRSF2*, *ASXL1*, and *U2AF1* are associated with a poor prognosis [29,35]. Somatic mutations in DNA methyltransferase 3A (*DNMT3A*), which involve DNA methylation, result in significant epigenetic changes and alterations in gene expression [31]. *RAS/CBL* mutations are linked to resistance to ruxolitinib. In patients with myelofibrosis, abnormal immature megakaryocytes in the bone marrow display reduced *GATA1* protein expression and secrete a range of pro-inflammatory cytokines (such as IL-1ß, TGF-ß) and growth factors (including b-FGF, PDGF, and VEGF). These inflammatory changes contribute to the development of bone marrow fibrosis and osteosclerosis [36].

The diagnosis of PMF involves comprehensive clinical and laboratory evaluations based on the WHO-HEM5 classification and International Consensus Classification (ICC) criteria from 2022 [2,37]. The updated diagnostic criteria place a stronger emphasis on integrating morphological, clinical, and genomic data to define disease entities. This includes the presence of *JAK2*, *CALR*, or *MPL* mutations, which are found in about 90% of PMF patients. Two major prognostic models have been developed to enhance the accuracy of predicting outcomes and guiding treatment planning for PMF by incorporating clinical, molecular, and karyotype data (Table 3): the Mutation-Enhanced International Prognostic Scoring System (MIPSS70/v2) and the Genetically Inspired Prognostic Scoring System (GIPSS) [38]. The GIPSS is a simpler model that relies solely on genomic markers, such as CALR, MPL, JAK2, and other high-mutation risk (HMR) genes, including *ASXL1*, *EZH2*, *SRSF2*, *IDH1*, *IDH2*, and *U2AF1* Q517, as well as specific karyotypes. Based on the GIPSS score, PMF patients can be categorized into favorable, intermediate, and unfavorable groups. In contrast, MIPSS70 is a more comprehensive approach that incorporates genetic mutations, cytogenetic data, and clinical information, allowing for better discrimination, particularly for those in the GIPSS intermediate-risk group [39,40]. Based on the MIPSS70 score, PMF patients can be further categorized into five risk groups: very high risk (median survival, 1.8 years), high risk (4.1 years), intermediate risk (7.7 years), low risk (16.4 years), and very low risk (median survival not reached). Both MIPSS70 and GIPSS can be easily computed using online tools.

Cytogenetic categories:

- Very high risk: single or multiple abnormalities of −7, i(17q), inv(3)/3q21, 12p−/12p11.2, 11q−/11q23, or other autosomal trisomies not including +8/+9 (eg, +21, +19).
- Favorable: normal karyotype or sole abnormalities of +9, 13q−, 20q−, chromosome 1 translocation/duplication, or sex chromosome abnormality including −Y.
- Unfavorable: all other abnormalities.

Risk Groups:

- High risk: GIPSS > 2; MIPSS70+ > 4.
- Intermediate risk: GIPSS 1–2.
- Low risk: GIPSS = 0; MIPSS70+ 1–4.

## 3. Molecular Characteristics of Ph-Negative MPN

Oncogenesis begins with critical mutations in essential genes, known as driver mutations. However, driver mutations alone do not guarantee clonal expansion [41,42]. Clonal expansion and evolution are characterized by genomic instability, which leads to the accumulation of diverse passenger mutations within the genome of oncogenic potential cells [41,42,43]. While the role of driver mutations in MPNs has been demonstrated in mouse models, the role of passenger mutations remains incompletely understood [44]. Additionally, the influence of age and gender on MPNs is notable. For instance, the *JAK2* V617F mutation can occur at any age, but MPNs are rare before the age of 50. After age 60, the incidence of MPNs is higher in men than in women [44,45].

### 3.1. Driver Mutations

#### 3.1.1. JAK (Janus Kinase)

The JAK2 mutation is one of the most frequent mutations identified in Ph-negative MPNs. JAK2 is associated with several receptors, including the erythropoietin receptor (EPOR) critical for the regulation of red blood cell production, the thrombopoietin receptor (MPL) essential for platelet production regulation, and various cytokine receptors such as the granulocyte colony-stimulating factor receptor (G-CSFR) and interleukin-3 receptor (IL-3R). The activation of *JAK2* initiates downstream signaling pathways, particularly the JAK/STAT pathway, which plays crucial roles in processes such as cell growth, development, differentiation, histone modifications, and the inflammatory response [46]. The *JAK2* V617F mutation is a dominant gain-of-function mutation in the JH2 pseudokinase domain, which normally regulates autoinhibition, leading to the continuous activation of the JAK/STAT pathway. The allele burden of *JAK2* V617F correlates directly with disease severity, as demonstrated in mouse models [47]. In humans, a higher quantitative *JAK2* V617F allele burden is associated with increased polycythemia and a higher rate of fibrotic transformation in patients with PV [48]. Furthermore, differential STAT signaling associated with *JAK2* V617F can lead to distinct clinical phenotypes [49]. The *JAK2* V617F mutation activates both *STAT5* and *STAT1* signaling pathways. In ET, *STAT1* activation constrains erythroid differentiation while promoting megakaryocytic differentiation [49]. Conversely, in PV, a reduced *STAT1* response coupled with an increased *STAT5* response to *JAK2* V617F removes the inhibitory effect on erythropoiesis, favoring the development of overt erythrocytosis [49].

Approximately 95% of patients have *JAK2 V617F* mutations (exon 14), while insertions and deletions in *JAK2* exon 12, such as the most common N542_E543del, occur in about 4% of cases [50,51]. Patients with *JAK2* exon 12 mutations exhibit distinct characteristics, including a younger age, higher hemoglobin levels, and lower platelet and reduced white blood cell counts at diagnosis [52]. However, patients with *JAK2* V617F and *JAK2* exon 12 mutations have a similar risk of thrombosis, progression to myelofibrosis or leukemia, and mortality [53].

In ET, the *JAK2* V617F mutation is present in approximately 50% of patients and is associated with higher hemoglobin levels, lower platelet counts, elevated leukocyte counts, and increased rates of venous thrombosis compared to ET patients without *JAK2* mutations [54]. In PMF, the *JAK2* V617F mutation is found in about half of the patients and is linked to higher neutrophil and platelet counts at diagnosis, a lower likelihood of transfusion dependence, and potentially poorer survival compared to other mutations [55,56].

#### 3.1.2. CALR (Calreticulin)

*CALR* is a calcium-binding protein involved in calcium storage, transcription regulation, and functioning as an endoplasmic reticulum (ER) chaperone. The most common *CALR* variants include type 1, a fifty-two-base pair deletion that disrupts calcium binding and activates the IRE1α/XBP1 response [57] and type 2, a five-base pair insertion that causes frameshifts and novel C-terminal proteins. Type 1-like and type 2-like mutations share structural features, leading to similar frameshifts and novel peptides. The loss of the C-terminal KDEL sequence, responsible for ER retrieval, shifts mutant *CALR* to the ER-Golgi intermediate and Golgi compartments [58]. Mutant *CALR* can bind to *MPL*, activating the *JAK2/STAT5* pathway independently of thrombopoietin. This dysregulates megakaryopoiesis and causes thrombocytosis [59]. *CALR* exon 9 mutations are detected in approximately 20–30% of patients with ET and PMF [60]. Type 1 mutations are more frequently observed in PMF, while type 2 mutations are predominantly associated with ET [61,62]. *CALR*-mutated ET is characterized by a younger age, male sex, higher platelet count, lower hemoglobin, lower leukocyte count, and lower thrombosis risk compared to *JAK2*-mutated ET [63]. However, *CALR* mutations do not affect OS or the risk of myelofibrotic or leukemic transformation [64].

In PMF, patients with type 1 *CALR* show improved OS compared to those with type 2 CALR mutations [65]. Patients with ET or PMF harboring type 1 mutations exhibit improved OS and a lower risk of thrombosis compared to those with the *JAK2* mutation [66].

#### 3.1.3. MPL (Myeloproliferative Leukemia Virus Oncogene)

MPL is a transmembrane protein with extracellular cytokine binding and intracellular signaling domains. When thrombopoietin (TPO) binds to MPL, it induces receptor dimerization, activating the JAK/STAT pathways, which are crucial for megakaryocyte growth and platelet production [67,68,69]. *MPL* mutations (exon 10) are reported in about 5–8% PMF and 1–4% of ET cases [70]. ET patients with *MPL* mutations, compared to those with V617F+ mutations, typically have lower hemoglobin, higher platelet counts at diagnosis, and elevated serum erythropoietin levels [70]. Triple-negative PMF is associated with inferior OS and leukemia-free survival (LFS) [71]. Table 4 summarizes the prognostic impact of the driver mutations in Ph-negative MPN.

### 3.2. Passenger Mutations

Passenger mutations are genetic alterations that occur in cancer cells but most are often considered byproducts of the genetic instability present in cancer cells, not directly contributing to the cancer’s growth or progression [41]. However, recent studies using NGS have identified some passenger mutations that are involved in epigenetic modulation in MPNs, such as *TET2*, *DNMT3A*, and *ASXL1* [73]. These passenger mutations might play a role in MPN clonal evolution and are therefore better referred to as co-mutations. Although their exact roles in MPN are still being defined, *ASXL1* mutations are commonly found in *CALR*-mutant MPN patients and *TET2* mutations are more frequent in *JAK2* V617F-mutated MPN patients. *TET2*-mutated MPN patients display a distinct gene expression signature compared with *TET2*-WT patients [74]. The loss of *TET2* function provides a strong competitive advantage to *JAK2* V617F-mutant hematopoietic stem cells. Conversely, the addition of *TET2* mutations exacerbates *JAK2* V617F-induced disease, causing prolonged leukocytosis, splenomegaly, extramedullary hematopoiesis, and slightly shorter survival [74,75]. *TET2* mutations can also modify DNA hydroxymethylation or methylation at specific sites in MPN cells, potentially playing a role in the pathogenesis of MPN.

### 3.3. Genomic Variation and Epigenetic Modification

Chromosome alterations in MPNs are diverse and vary depending on the specific subtype. These alterations are among the most common copy number variations (CNVs), offering significant growth advantages for quicker clonal expansion and playing a key role in the progression and prognosis of these diseases. Chromosome changes are most prevalent in PMF at 55%, followed by PV at 20%, and are infrequent in ET at only 5% [76,77]. A 4q loss is associated with *TET2* mutations, and recurrent 11q CN-LOH is observed across various MPNs. A 1q gain is most prevalent in PV and ET cases, particularly those with the *JAK2* V617F mutation, whereas deletion of 17p is more common in PMF patients associated with dysregulation of the *TP53* pathway [78]. 5q deletions are largely overlapped between MDS, MPN, and MDS/MPN [79]. 20q deletions are frequently seen in all MPNs. A 9p gain or trisomy 9 is specific to PV and secondary myelofibrosis [80]. Trisomy 8, in contrast, is commonly seen in PMF patients.

Epigenetic changes in MPNs involve reversible modifications to DNA- and RNA-associated proteins that impact gene activity without altering the DNA sequence. These changes include alterations in DNA methylation, histone modification, and microRNA expression patterns. Increasing evidence indicates that these epigenetic changes can collectively influence gene expression and potentially contribute to MPN pathogenesis. Clinical trials evaluating HDAC inhibitors and DNA methyltransferase inhibitors for the treatment of patients with MPNs are currently underway [81]. Some epigenetic changes may be linked to the transformation of MPNs [82]. *DNMT3A* encodes an enzyme responsible for DNA methylation, and *TET2* involves DNA demethylation. Co-mutations in *DNMT3A* and/or *TET2* genes result in DNA methylation abnormalities. The altered DNA methylation patterns can lead to DNA breakage, causing a loss of genetic materials (del5q, del7q, and del17p) and/or duplications (8q and *MYC*). In addition, epigenetic changes can also cause an abnormal telomere length, impacting the disease expression [83]. Chromatin architectures differ significantly between the ligand-induced activation of the JAK/STAT pathways and the chronic constitutive activation of STAT proteins [84]. The mutant *JAK2* V617 protein can affect the chromatin landscape independently of STAT protein interactions. The *JAK2* V617F mutant phosphorylates protein arginine methyltransferase 5 (*PRMT5*) and impairs *PRMT5* histone methylation, enhancing myeloproliferation [85]. These findings demonstrate a crosstalk between oncogenic kinases and epigenetic regulation at the molecular level, which may provide new insights into potential novel treatment strategies for MPNs [85].

### 3.4. Genetic Inheritance and Germline Predisposition

The majority of MPNs are acquired through somatic mutations in driver and passenger genes. However, germline susceptibility does exist. For example, germline mutations in the *JAK2* JH1 and JH2 domains can lead to hereditary thrombocytosis, which mimics sporadic ET and may also involve myelofibrotic transformation [86,87,88]. Familial thrombocytosis (FT) is a rare autosomal-dominant disorder, primarily caused by activating mutations in the thrombopoietin (*THPO*) gene and germline mutations in the myeloproliferative leukemia (*MPL*) virus oncogene [89,90].

Primary familial and congenital polycythemia (PFCP) is a rare autosomal-dominant disorder characterized by congenital erythrocytosis, where mutations in the erythropoietin receptor (EpoR) have been identified [91]. These mutations produce a truncated erythropoietin receptor protein, causing erythroid progenitors to become hypersensitive to erythropoietin. As a result, patients with these mutations exhibit elevated hematocrit levels and low erythropoietin levels, without the additional somatic driver mutations or leukemic transformation propensity seen in sporadic PV. Chuvash polycythemia, an autosomal recessive disorder endemic to the mid-Volga River region, is associated with a mutation in the *VHL* gene, specifically an arginine-to-tryptophan substitution at amino acid residue 200 (Arg200Trp) [92]. The VHL protein regulates the ubiquitination and degradation of hypoxia-inducible factor 1 subunit alpha (*HIF1* alpha). Additionally, a mutation in the transmembrane domain of the *MPL* protein (*MPL*-S505N) was discovered in a family with autosomal-dominant thrombocytosis [89]. Interestingly, this same mutation has also been found in cases of sporadic ET [70].

## 4. Pivotal Clinical Trials and Therapeutic Update

### 4.1. Chronic Myeloid Leukemia (CML)

Chronic myeloid leukemia (CML) is the only Ph-positive MPN. Since the approval of imatinib in 2001, five additional BCR-ABL TKIs have been approved for the treatment of CML. These include the first-generation TKI imatinib, second-generation TKIs dasatinib, nilotinib, and bosutinib, and third-generation TKIs ponatinib and asciminib. Newer generation TKIs have also been approved for treating CML patients who have failed firstline imatinib therapy, including those with the T315I mutation. Despite significant advances in TKI therapy, allogeneic stem cell transplantation remains a crucial curative option for patients who do not respond adequately to TKIs or for those experiencing accelerated or blast crisis phase progression.

The selection of a TKI for CML should be individualized based on factors such as the patient’s risk score, toxicity profile, age, ability to tolerate therapy, and the presence of comorbid conditions. For most patients, the primary goals of treatment are to induce and maintain remission, prevent disease recurrence and progression, and improve quality of life and survival. For some patients, achieving treatment-free remission (TFR) is an important goal, which becomes possible with newer generation TKIs. While these newer generation TKIs offer deeper and faster cytogenetic and molecular responses, there are no significant survival differences among the approved TKIs for CML. A retrospective analysis of five clinical trials found no significant differences in 5-year OS among patients treated with imatinib 400 mg, imatinib 800 mg, dasatinib, or nilotinib [93]. Additionally, a systematic review and network meta-analysis comparing the efficacy and safety of TKIs in CML patients concluded that newer TKIs (nilotinib, dasatinib, bosutinib, and ponatinib) do not show a significant survival advantage over imatinib [94].

TFR refers to a sustained deep molecular response, typically indicated by a 4 or 4.5 log reduction in the *BCR-ABL1* gene transcript levels, maintained for a defined period (often at least 2 years) following the cessation of TKI therapy in CML patients. Several TKI stop studies have shown that nearly 50% of CML patients can safely discontinue TKIs and achieve TFR. This enables eligible patients to stop lifelong TKI treatment, minimizing drug-related adverse effects and financial strain while preserving disease control. Most relapses after stopping TKIs occur within 6 to 12 months, but many patients who lose molecular response can regain it rapidly upon restarting therapy. The feasibility and safety of TFR have been confirmed through multiple clinical trials, and current guidelines have recognized TFR as a key treatment objective [95].

#### 4.1.1. FDA Approved TKIs (Table 5)

(a). Imatinib (Gleevec)

**Table 5:** Long-term outcomes of pivotal clinical trials for newly diagnosed CML.

Trial	MMR (%) (Cumulative)	MR 4/4.5 (%) (Cumulative)	PFS (%) (at the End of Study)	OS (%) (at the End of Study)	Activity Against T315I Mut	Follow-Up
**IRIS** (IM vs. IFN)	93.1 vs. NA	63.2 vs. NA	79.6 vs. 56.6	82.3 vs. NA (HR 0.74)	No	11 years [96]
**DASISION** (DAS vs. IM)	76 vs. 64	42 vs. 33	85 vs. 86	91 vs. 90	No	5 years [97]
**ENESTnd** (NIL 300 vs. IM)	77.7 vs. 62.5	61 vs. 39.2	86.2 vs. 87.2	87.6 vs. 88.3	No	10 years [98]
**BEFORE** (BOS vs. IM)	74.4 vs. 63.3	58.2 vs. 48.1 (MR4) 47.4 vs. 36.6 (MR 4.5)	NA	94.5 vs. 94.6	No	5 years [99]
**PACE (phase II)** (Ponatinib)	Not yet approved for newly diagnosed CML.				yes	
**ASC4FIRST** (Asciminib vs. 2G TKI)	74.1 vs. 52.8	NA	NA	NA	yes	2 years [100]

**Note:** The cumulative response analysis was conducted at the completion of the study for MMR with *BCR::ABL1* transcript levels ≤ 0.1% on the International Scale (IS), MR4 with *BCR::ABL1* transcript levels ≤ 0.01% on the IS, and MR4.5 with *BCR::ABL1* transcript levels ≤ 0.0032% on the IS. Additionally, the analysis included PFS and OS.

Imatinib, also known as Gleevec, was the first drug approved in 2001 for the treatment of CML across all phases: CP, AP, and BP. The landmark IRIS trial (International Randomized Study of Interferon vs STI571) established the efficacy and safety of imatinib for newly diagnosed CML-chronic phase patients. The trial demonstrated significantly higher rates of complete cytogenetic response (CCyR) with imatinib compared to interferon plus cytarabine (IFN/Ara-C) at 18 months, with CCyR rates of 76.2% for imatinib versus 14.5% for IFN/Ara-C [101].

The 11-year follow-up confirmed the sustained efficacy of imatinib over time, with no unacceptable late toxic or cumulative effects. The estimated OS rate was 83.3% for patients receiving imatinib compared to 78.8% for those on interferon. The 10-year cumulative CCyR and major molecular response (MMR) rates were 91.8% and 93%, respectively. Progression to the accelerated phase or blast crisis during this study was infrequent in imatinib-treated patients (6.9% vs. 12.8%) [96].

Common side effects include nausea, vomiting, diarrhea, muscle pain, and rash. Severe side effects such as fluid retention, liver problems, and heart issues were uncommon and most frequently occurred during the first year of treatment [101].

(b). Dasatinib (Sprycel)

Dasatinib, marketed as Sprycel, is an oral TKI that is 350 times more potent than imatinib in vitro [102]. In addition to inhibiting BCR-ABL, dasatinib also suppresses SRC family kinases (*SRC*, *LCK*, *YES*, *FYN*), as well as c-*KIT*, *EPHA2*, and *PDGFRβ* kinases. Dasatinib received accelerated approval in 2006 and full approval on 26 May 2009, for the treatment of CML in all phases (CP, AP, and BP) after disease progression or intolerance to prior therapies, including imatinib. The efficacy, safety, and dose optimization of dasatinib were evaluated in a phase 3 randomized, open-label dose-optimization study (CA180-034) that included 670 patients who were resistant or intolerant to imatinib [103]. By 22 months, 63% of patients achieved a sustained major cytogenetic response (MCyR). The seven-year follow-up showed that the rates for MMR, progression-free survival (PFS), and OS were 46%, 42%, and 65%, respectively, at dasatinib 100 mg once daily, and similar across doses [104]. Improved PFS and OS rates were reported in patients who achieved *BCR-ABL1* ≤ 10% at 3 and 6 months [104]. No new safety signals were identified, with the incidence of drug-related pleural effusion at 28% and arterial ischemic events at 4%. These data support the long-term efficacy and well-established safety profile of dasatinib for patients after imatinib failure.

Dasatinib received approval for the frontline therapy of newly diagnosed CML in the chronic phase on 28 October 2009, based on the results from the DASISION trial, which compared the efficacy and safety of dasatinib with imatinib in newly diagnosed CML-CP patients [105]. Dasatinib showed superior efficacy as a frontline therapy. By 12 months, 83% of patients achieved CCyR with dasatinib compared to 72% with imatinib. MMR rates were 46% for dasatinib versus 28% for imatinib [105]. At the five-year mark, dasatinib continued to show higher response rates for newly diagnosed CML. The CCyR rate was 83% for dasatinib versus 78% for imatinib; MMR rates were 76% versus 64%, and the molecular response (MR) 4.5 rate (4.5 log reduction or BCR-ABL ≤ 0.0032%) was 42% versus 33%, respectively [97]. Despite improvements in cytogenetic and major molecular responses, the five-year PFS rates (85% for dasatinib and 86% for imatinib) and OS rates (91% for dasatinib and 90% for imatinib) were similar [97]. However, fewer patients on dasatinib transformed to accelerated or blast-phase CML compared to those on imatinib (4.6% vs. 7.3%). These results were confirmed by other multicenter randomized studies [106]. More recently, a lower dose of dasatinib (50 mg daily) was found to be just as effective and better tolerated as the standard dose for newly diagnosed CML patients. At this lower dose, the 12-month MMR rate was 81%. The cumulative incidence for MR4, MR4.5, and complete molecular response (CMR) within one year were 63%, 53%, and 46%, respectively. After a median follow-up of five years, the event-free survival (EFS) and OS rates were 97% and 98%, respectively [107].

The safety profile of dasatinib has remained consistent throughout five years of follow-up. Pleural effusions and hematologic toxicity occurred more frequently with dasatinib (19% vs. <1%), and rare cases of pulmonary hypertension were reported (1–2%) in patients treated with dasatinib. The discontinuation rate due to pleural effusion was 6% in the dasatinib arm. Other common side effects include low white blood cell counts, low blood platelets, anemia, swelling, rash, and diarrhea. Severe side effects can include bleeding, pulmonary edema, heart failure, and prolonged QT syndrome.

(c). Nilotinib (Tasigna)

Nilotinib, marketed as tasigna, is a second-generation TKI that is structurally similar to imatinib but has 30–50 times greater affinity for the ATP binding site on BCR-ABL1 in vitro [108]. Nilotinib received accelerated approval on 29 October 2007 for the treatment of CML in both chronic and accelerated phases after imatinib failure or intolerance. The approval was based on the results of a single-arm, open-label, phase 2 clinical trial that demonstrated major cytogenetic and hematologic responses [109].

On 18 June 2010, nilotinib (Tasigna) received full FDA approval for the frontline treatment of newly diagnosed CML in the chronic phase (CML-CP). This approval was based on the results of the ENESTnd trial, a phase 3 randomized study evaluating the efficacy and safety of nilotinib in newly diagnosed CML-CP patients [110]. At 12 months, the rates of MMR for nilotinib (44% for the 300 mg dose and 43% for the 400 mg dose) were nearly twice those for imatinib (22%). The rates of CCyR by 12 months were also significantly higher for nilotinib (80% for the 300 mg dose and 78% for the 400 mg dose) compared to imatinib (65%). Additionally, nilotinib induced earlier and higher responses at 3 months compared to imatinib and significantly reduced the risk of progression to the accelerated phase or blast crisis, establishing nilotinib as a superior therapeutic option to imatinib for treating newly diagnosed CML [110]. By 5 years, more than half of the patients in each nilotinib arm achieved a molecular response of 4.5 (MR4.5), compared to 31% in the imatinib arm [111]. By 10 years, the cumulative incidence of MMR reached 77.7% for patients receiving 300 mg nilotinib twice daily, compared to 62.5% for those on imatinib (*p* < 0.0001). Additionally, the cumulative rates of MR4.5 were 61% for nilotinib and 39.2% for imatinib. Despite the superior response rates, there was no significant difference in the estimated 10-year OS between patients treated with nilotinib and imatinib [98]. Overall, the long-term follow-up continues to support the use of nilotinib 300 mg BID as frontline therapy for CML, especially for those aiming for treatment-free remission [98].

Adverse events were similar between nilotinib and imatinib, although nilotinib was associated with higher cumulative rates of cardiovascular events, including pleural effusion, pericardial effusion, pulmonary edema, and QT prolongation [98]. Baseline Framingham cardiovascular risk scores and existing cardiovascular morbidities were predictive of patients’ risk of developing a cardiovascular event during treatment.

(d). Bosutinib (Bosulif)

Bosutinib, marketed as Bosulif, is a potent dual SRC/ABL kinase inhibitor. It received approval on 4 September 2012, for treating patients with CML in chronic, accelerated, or blast phases after the failure of at least one prior TKI therapy. The approval was based on phase 1/2 study results that demonstrated durable efficacy and a tolerable safety profile [112,113]. Following its market release, the phase 4 BYOND trial was conducted for further evaluation, with the final results published in 2024 [114,115]. By 5 years, 81.1% of patients maintained CCyR, 71.8% maintained MMR, and 59.7% maintained a molecular response of 4 (MR4). The five OS rate was 88.3%. Overall, these studies confirmed the long-term efficacy of bosutinib for the treatment of patients with CML in the second line.

On 19 December 2017, bosutinib received approval for treating patients with newly diagnosed CML based on the results of the BFORE trial, a phase 3 randomized study that evaluated the efficacy and safety of bosutinib in newly diagnosed CML-CP patients [116]. The final results of the 5-year follow-up were published in 2022 [117]. At 12 months, the CCyR rate was 77% for bosutinib compared to 66% for imatinib. The MMR rate was 41% for bosutinib versus 32.7% for imatinib. By two years, 61% of patients in the bosutinib arm versus 51% in the imatinib arm remained in MMR. By five years, the cumulative MMR rate was 74.4% for bosutinib compared to 63.3% for imatinib. The MR4 rate was 58.2% for bosutinib versus 48.1% for imatinib, and the MR4.5 rate was 47.4% for bosutinib versus 36.6% for imatinib. Despite the improved molecular response rates, there was no significant difference in the 5-year OS rates between the two treatment arms (94.5% for bosutinib versus 94.6% for imatinib) [99,117]. During treatment, the transformation rates to the accelerated or blast phase were similar in both bosutinib- and imatinib-treated patients [99].

The most common side effects of bosutinib are nausea, vomiting, diarrhea, stomach pain, and rash. Severe toxicities may involve bleeding or bruising, liver problems, heart issues, fluid retention, and low blood cell counts. Patients treated with bosutinib experienced an increase in cardiac, effusion, renal, and vascular issues [99]. The most common causes of permanent treatment discontinuation were elevated ALT levels (7.7%) with bosutinib and increased lipase levels (4.1%) with imatinib [99].

(e). Ponatinib (Iclusig)

Ponatinib, also known as Iclusig, is a third-generation *BCR-ABL1* kinase inhibitor against all known *BCR-ABL1* mutations, including the T315I mutation. It received FDA approval for the treatment of CML in December 2012, based on the results of the phase II PACE (Ponatinib Ph+ ALL and CML Evaluation) trial. This trial evaluated ponatinib in patients with CML who had failed prior TKI therapy, including those with the T315I mutation [118]. The results showed that ponatinib induced a MCyR in 55% of patients, a CCyR in 46% of patients, and an MMR in 34% of patients at 12 months [118]. At the 5-year update, 60% of patients achieved MCyR, 40% achieved MMR, and 24% achieved MR4.5. Additionally, 82% of the responders remained at MCyR for 5 years, indicating that ponatinib induced a durable and clinically meaningful response in this heavily pre-treated patient population [119]. The two-year PFS and OS rates were 68% and 85%, respectively [120].

The OPTIC trial, a randomized open-label multicenter study, evaluated ponatinib in heavily pre-treated CML-CP patients, including those with the T315I mutation [121]. The study involved three cohorts receiving ponatinib at starting doses of 45 mg, 30 mg, and 15 mg once daily. All starting doses showed clinical benefit, with the optimal benefit/risk outcomes observed at 45 mg, which was later reduced to 15 mg upon achieving a response [121]. At 12 months, 44.1% of the 45 mg cohort patients achieved the primary endpoint of <1% *BCR-ABL1* IS. Both the PACE and OPTIC trials demonstrated ponatinib’s efficacy in heavily pre-treated CP-CML patients, with the OPTIC trial specifically addressing dose optimization to balance efficacy and safety. At 24 months, 46% of patients in the PACE trial and 57% in the OPTIC trial achieved a BCR-ABL1 response of ≤1%. PFS rates were 68% in the PACE trial and 80% in the OPTIC trial. OS rates were 85% in the PACE trial and 91% in the OPTIC trial [120].

Both trials reported serious side effects, including arterial thrombosis, heart attack, and stroke. Currently, ponatinib (Iclusig) has not been approved for the frontline treatment of newly diagnosed CML.

(f). Asciminib (Scemblix)

Asciminib is a STAMP inhibitor that specifically targets the *ABL* myristoyl pocket in the BCR-ABL kinase enzyme. This mechanism distinguishes it from other TKIs, allowing it to be effective against both native and mutated *BCR-ABL1*, including certain mutations that confer resistance to other treatments [122]. On 29 October 2021, asciminib received accelerated approval for treating CML-CP after the failure of two or more prior TKIs, based on the results of the pivotal phase III ASCEMBL study and the phase 1 dose-escalation study [122,123]. In the ASCEMBL trial, asciminib demonstrated a superior MMR rate compared to bosutinib (25% vs. 13%) at 24 weeks. Asciminib was well-tolerated, with significantly lower discontinuation rates due to toxicity (7% vs. 25%). With 20 months of follow-up, the median duration of MMR has not yet been reached. The approval included patients with the T315I mutation. The phase I CABL001X2101 (NCT02081378) study showed a 42% MMR rate at 24 weeks and a 49% MMR rate at 96 weeks in patients with T315I mutations [124].

The ASC4FIRST study, a phase III, multicenter, open-label randomized trial, evaluated the efficacy and safety of asciminib versus imatinib or second-generation TKIs (2G TKIs) in patients with newly diagnosed CML in the chronic phase (CML-CP) [125]. At 12 months, 66% of patients treated with asciminib achieved an MMR compared to 57.8% of patients treated with second-generation TKIs. Based on these results, asciminib received accelerated approval on 29 October 2024 for the frontline treatment of newly diagnosed CML-CP. An additional one-year follow-up, presented at ASH in December 2024, demonstrated that asciminib continued to show superior efficacy in patients with newly diagnosed CML. At 96 weeks, the MMR rate was 74.1% in the asciminib arm compared to 52% for all TKIs combined. Specifically, the MMR rate was 76.2% for asciminib versus 47.1% for imatinib, and 72% for asciminib versus 56.9% for second-generation TKIs (nilotinib, dasatinib, and bosutinib) [126]. The cumulative incidence of deep molecular response (MR4, MR4.5) was also higher with asciminib than with all other TKIs, and it increased over time. Regarding the safety and tolerability profile, the data continue to favor asciminib, with a higher rate of discontinuation due to toxicity observed with imatinib and second-generation TKIs. Arterial-occlusive events were infrequently observed in the study, with 2% of all grads for asciminib, 2/9% for 2G TKIs, and 0% for imatinib. The post-baseline mutation analysis by NGS revealed a treatment-emergent BCR-ABL gene mutation in the myristoyl pocket that may confer resistance to asciminib.

#### 4.1.2. Novel Agents: Olverembatinib and Vodobatinib

Olverembatinib, also known as HQP1351, is a third-generation BCR-ABL1 TKI developed to treat CML in patients who are resistant or intolerant to multiple TKIs, including ponatinib and asciminib. In a phase 1/2 trial, olverembatinib showed significant antileukemic activity, a favorable pharmacokinetic profile, and was well tolerated in patients with heavily pre-treated chronic-phase CML with or without T315I variants, including those who had previously failed ponatinib and/or asciminib, achieving high rates of MCyR and MMR [127,128]. In the updated pivotal phase 2 study, patients achieved a CCyR rate of 53.6% and an MMR rate of 40% for ponatinib-resistant cases, and a CCyR rate of 37.5% and an MMR rate of 30% for asciminib-resistant cases [129]. These findings led to the drug being included in the latest NCCN guidelines for CML management. Phase III trials are being planned to confirm these results. The potential mechanism of olverembatinib to overcome resistance to ponatinib and asciminib may be due to its unique ATP-site binding, with high efficacy against broad range of *BCR::ABL1* mutations, including T315I and certain compound mutations, where other agents often fail because of their specific resistance profiles [130].

**Vodobatinib** is another novel, orally administered selective TKI that targets *BCR::ABL1* and c-Abl kinases. It is under clinical investigation for the treatment of Ph-positive CML resistant to multiple prior TKIs, including ponatinib and asciminib. In a recent phase 1/2 trial, vodobatinib demonstrated clinically meaningful antileukemic activity and a tolerable safety profile in heavily pre-treated CML patients [131].

#### 4.1.3. Impact of Cancer Gene Variants on Treatment Response

Genome-wide mutational analyses identified cancer gene variants in CML at diagnosis, with ASXL1 being the most frequently mutated gene (9%) in patients with CML at diagnosis [132]. This mutation is associated with increased treatment failure and kinase domain mutation acquisition. Studies have found that the presence of mutated *ASXL1* at diagnosis negatively impacts 2G-TKI-treated patients, resulting in a lower major molecular response, reduced failure-free survival, and an increased rate of kinase domain mutation acquisition. For asciminib-treated patients, the presence of *ASXL1* variants at diagnosis led to even higher failure rates compared to 2G-TKIs. By two years, 29% of all patients with *ASXL1* variants at diagnosis developed a kinase domain mutation, compared to 3% of patients without these variants. However, the *ASXL1* variant allele frequency or variant location was not predictive of the development of kinase domain mutations.

#### 4.1.4. Evolving Strategies for CML Treatment: Balancing Efficacy and Safety

The therapeutic landscape of CML has been revolutionized by TKIs. With six highly effective TKIs already approved and more on the horizon, selecting the most suitable TKI has become a sophisticated practice, requiring a careful balance of efficacy, safety, and understanding of disease biology. Low-dose TKIs, such as dasatinib 50 mg daily instead of the standard 100 mg daily, appear to be just as effective and safer in both frontline and laterline therapy for CML [107]. Reducing the TKI dosage after achieving MMR is another proven strategy for the effective and safer long-term treatment of CML, while maintaining the same deep MMR, PFS, and OS. True resistance to TKIs, including imatinib, is uncommon. Although the discontinuation rate of early-generation TKIs was reported to be high, most discontinuations were due to toxicity rather than true drug resistance [133]. With more dose-optimization data available, a dose reduction rather than changing TKIs is preferred. The notion of using the best TKI upfront is arguable without considering cost and/or long-term safety. While newer generation TKIs yield faster and deeper responses compared to generic imatinib, there are no significant differences in PFS and OS across all currently approved TKIs [134]. Safety data for TKIs should be monitored for at least 7–8 years for CML treatment [135]. For example, longer follow-ups showed that nilotinib was associated with an increased risk of cardiovascular events, such as severe hypertension and arterial-occlusive events. Asciminib was the newest TKI approved for frontline treatment for CML. There is a strong inclination to use asciminib because of its higher efficacy and early safety data; however, its long-term safety profile is yet to be fully established. Many toxicities of TKIs do not necessitate a change in treatment unless the patient experiences prohibitive toxicity, including recurrent pleural effusion, pulmonary hypertension, arterial-occlusive or vaso-occlusive events, neurologic events, immune-mediated events like pneumonitis, myocarditis, etc. Aggressively pursuing TFR to achieve PCR < 0.01% may be counterproductive and could potentially cause more harm than good. CML patients with PCR levels between 1 and 10% can still lead a normal life during the chronic phase [136].

### 4.2. Therapeutic Updates for PV, ET, and PMF

#### 4.2.1. Polycythemia Vera (PV)

The primary goal in treating PV is to reduce the risk of thrombotic complications and alleviate disease-related symptoms by normalizing blood counts. Regular phlebotomy remains the cornerstone treatment for all PV patients, aiming to maintain a hematocrit value below 45%. Criteria for cytoreduction include age over 60 years and/or a history of thrombosis or vascular events such as stroke. Hydroxyurea (HU) is the firstline treatment for cytoreduction. However, up to 25% of patients on hydroxyurea will eventually develop resistance or intolerance, which leads to an increased risk of mortality and leukemic transformation in this population.

**(a). Ruxolitinib:** Also known as Jakafi, it was approved for the treatment of PV patients after failure or intolerance to hydroxyurea. Two pivotal clinical studies, the RESPONSE and MAJIC trials, assessed the efficacy and safety of ruxolitinib versus the best available therapy (BAT) in a secondline setting for PV patients after hydroxyurea failure [137]. The RESPONSE trial, a randomized, open-label, phase 3 study, enrolled 222 PV patients who were either resistant to or intolerant of hydroxyurea. This study demonstrated that ruxolitinib was superior in controlling hematocrit without phlebotomy and reducing spleen size compared to BAT [137]. The MAJIC-PV trial, a randomized, open-label, phase 2 study, had a similar design to the RESPONSE trial. It showed that ruxolitinib achieved a higher complete response rate compared to BAT (43% vs. 26%) and significantly reduced the risk of major thrombosis, hemorrhage, transformation, or death [138].

**(b). Interferon:** Interferon-alpha works by modulating the immune system and inhibiting the proliferation of malignant cells in bone marrow. Studies have shown that interferon-alpha can achieve high response rates in PV patients, particularly those who are resistant or intolerant to other treatments like hydroxyurea [139]. Ropeginterferon alfa-2b-njft, also known as besremi, was the first FDA-approved interferon therapy for use in PV patients, regardless of their treatment history. Besremi was evaluated in a phase 3 study for its efficacy and safety in patients with PV [140]. The trial results demonstrated robust and durable rates of complete hematologic response (CHR), defined as no phlebotomy in the past 2 months, hematocrit < 45%, leukocyte count ≤ 10 × 10^9^/L, and platelet count ≤ 400 × 10^9^/L. Long-term follow-up over 7.5 years showed zero thromboembolic events, no cases of acute myeloid leukemia, and only one case of myelofibrosis [141]. Besremi was also shown to decrease the JAK2 allelic burden and demonstrates intriguing potential for modifying the disease [142].

Pegylated interferon has not yet received FDA approval for the treatment of PV or ET, but it has been used off-label for these conditions. The efficacy and safety of pegylated interferon were evaluated in a phase 2, single-center, prospective trial presented at ASH 2024. The final analysis showed a high rate of hematologic response, with 85% of PV patients and 58% of ET patients achieving a complete hematologic response (CHR) [143]. Additionally, there was a significant reduction in the JAK2 allelic burden in 58% of PV patients (more than 50% reduction) [143].

**(c). Rusfertide:** In PV, hepcidin plays a crucial role in regulating iron homeostasis. New methods are being developed to decrease the need for phlebotomy by targeting hepcidin. Rusfertide (PTG-300) is an injectable hepcidin-mimetic peptide. By mimicking endogenous hepcidin, it restricts the availability of iron for erythropoiesis in a dose-dependent manner in healthy volunteers [144]. The REVIVE study, a phase II trial, evaluated the efficacy and safety of rusfertide in PV patients who require more frequent therapeutic phlebotomies. The trial demonstrated that rusfertide therapy effectively controlled erythrocytosis, maintained a hematocrit value of less than 45%, and reduced or eliminated the need for phlebotomy in some PV patients. While not yet approved, rusfertide represents a novel approach to controlling hematocrit and alleviating symptoms in PV patients [145]. To confirm the results of the REVIVE study, the VERIFY study, a phase 3, multicenter, global, randomized trial, was initiated in 2022 to compare the efficacy and safety of rusfertide versus placebo when added to ongoing therapy for PV [144].

**(d). Divesiran:** Presented at ASCO 2024, the SANRECO trial is an ongoing phase 1/2 study evaluating divesiran, a novel GalNAc-conjugated siRNA targeting TMPRSS6 (transmembrane protease, serine 6), in patients with PV. *TMPRSS6* negatively regulates hepcidin, the hormone responsible for controlling iron absorption and distribution. Inhibiting TMPRSS6 raises hepcidin levels and reduces iron delivery to the bone marrow, resulting in decreased erythropoiesis. Initial results from the SANRECO trial showed that new methods for modulating hepcidin, such as divesiran, nearly eliminated the need for phlebotomy in patients with PV [146].

#### 4.2.2. Essential Thrombocythemia (ET)

The primary goal of treating ET is to reduce the risk of thrombotic events by normalizing blood counts, while also improving quality of life and decreasing the risk of disease transformation into more serious conditions like myelofibrosis or acute myeloid leukemia. Low-dose aspirin is the cornerstone therapy for reducing the risk of thrombotic and vascular events, except for patients in the very low-risk group. For high-risk patients, cytoreductive therapy is recommended, although its benefit for intermediate-risk patients remains controversial. Hydroxyurea (HU) has been the mainstay treatment for ET patients when cytoreductive therapy is indicated. Anagrelide, a selective platelet-lowering agent, inhibits megakaryocyte maturation. Comparative trials have shown mixed results: the ANAHYDRET trial demonstrated the noninferiority of anagrelide to hydroxyurea for thrombotic event prevention in ET, while the UK-PT1 trial found hydroxyurea to be superior for reducing arterial thrombosis, serious hemorrhage, and myelofibrosis transformation, whereas anagrelide was associated with fewer venous thromboembolic events and higher rates of cardiovascular toxicity [147,148]. It is therefore generally reserved as a secondline agent for ET patients who are intolerant of or resistant to hydroxyurea. Interferon-α is commonly used off-label for ET patients, especially when hydroxyurea is contraindicated, such as during pregnancy. The Myeloproliferative Disorders Research Consortium (MPD-RC) 112 trial compared the efficacy and safety of pegylated IFN-a (PEG) directly with hydroxyurea in the treatment of high-risk PV and ET. The trial results showed that both PEG and HU were effective in inducing complete hematological response (CHR) at 12 months, with significant reductions in the JAK2 allelic burden observed in 58% of PV patients treated with PEG, compared to 43% with HU [149]. With a longer follow-up, PEG was more effective in normalizing blood counts (65% vs. 43%) and achieving a molecular response, while HU showed more histopathologic responses in ET patients [149]. Despite these differences, both agents did not significantly differ in limiting thrombotic events and disease progression in high-risk patients with ET/PV. Grade 3/4 adverse events were more common with interferon (46% vs. 28%).

#### 4.2.3. Primary Myelofibrosis (PMF) (Table 6)

For most low-risk or very low-risk PMF patients, observation is recommended rather than early treatment, as these patients have an estimated 10-year survival rate of 56–92%. For intermediate and high-risk patients, *JAK* inhibitors have been the cornerstone of treatment for symptom management, including reducing splenomegaly and providing symptom relief. While JAK inhibitors like ruxolitinib, fedratinib, and pacritinib have shown efficacy in reducing splenomegaly and symptom burden, they do not significantly alter disease progression and are often limited by hematologic toxicities. The only curative treatment for PMF is allogeneic stem cell transplantation (allo-HCT), which is typically reserved for high-risk PMF patients whose estimated 10-year survival is 0–13% [26]. A large multicenter retrospective study showed that patients with intermediate-1 (Int-1) or higher-risk myelofibrosis experienced a long-term survival benefit following allo-HCT, despite the higher risk of early transplant-related complications associated with this treatment [150]. Patients aged 65 years or older and male patients are likely to benefit more from allo-HCT compared to younger and female patients [151]. The post-transplantation clearance of driver mutations at day 30 is an independent predictor of disease relapse and survival, regardless of the underlying driver mutation [152].

**Table 6:** Pivotal clinicals for JAK2 inhibitors in patients with myelofibrosis.

JAK Inhibitor (Brand Name)	Targets	Clinical Trial	SVR35 at 24 Weeks	TSS50 at 24 Weeks	OS	Common Side Effect
Ruxolitinib (Jakafi)	JAK1, JAK2	**COMFORT-I** 1st line: Ruxolitinib vs. Placebo **COMFORT-II** 1st line: Ruxolitinib vs. BAT	41.9% vs. 0.7% 31.9% vs. 0%	45.9% vs. 5.3%	91.6% vs. 84.4% (HR = 0.5) NR vs. 4.1 yrs (0.52)	Thrombocytopenia, anemia, infections
Fedratinib (Inrebic)	JAK2	**JAKARTA** 1st line: Fedratinib vs. BAT **FREEDOM2:** 2nd line after ruxolitinib	47% vs. 1% 35% vs. 6%	40% vs. 9% 34.1% vs. 16.9%	NA NA	Dizziness, diarrhea, nausea, low platelet counts
Pacritinib (Vonjo)	JAK2, FLT3, IRAK1, CSF1R	**PERSIST-1** 1st line, Pacritinib vs. BAT **PERSIST** (prior ruxolitinib) 2nd line, Pacritinib vs. BAT	19% vs. 5% 22% vs. 3% 15% vs. 2% (once daily) 22% vs. 2% (twice daily)	32% vs. 14% 33% vs. 15% (prior ruxolitinib)	NA OS benefit in subgroup patient with spleen response	Bleeding, cardiac events
Momelotinib (Ojjaara)	JAK1, JAK2, ACVR1	**SIMPLIFY-1** 1st line: momelotinib vs. ruxolitinib **SIMPLIFY-2** (prior ruxolitinib) 2nd line: momelotinib vs. BAT	26.5% vs. 29% 6% vs. 7%	28.4% vs. 42.2% 26% vs. 6%	65.8% vs. 61.2% at 2 yrs	Dizziness, fatigue, diarrhea, liver problems

SVR35: the proportion of patients with a reduction of 35% or more in spleen volume from baseline; TSS50: the proportion of patients with reduction in TSS of 50% or more from baseline; JAK2 V617F values at baseline, measured in blood samples using allele-specific real-time quantitative PCR (RQ-PCR).

#### 4.2.4. JAK Inhibitors (Table 6)

**(a). Ruxolitinib:** Ruxolitinib was the first JAK inhibitor approved by the FDA for the treatment of intermediate or high-risk myelofibrosis in 2011. The efficacy and safety of ruxolitinib were evaluated in two randomized phase III trials, COMFORT-1 and COMFORT-2. The COMFORT-1 trial was a randomized, double-blind, placebo-controlled phase 3 study conducted in the United States, Australia, and Canada, enrolling 309 patients with intermediate-2 or high-risk myelofibrosis [153]. The primary endpoint was a reduction of 35% or more in spleen volume at 24 weeks (SVR35). Secondary endpoints included symptom improvement (measured by >50% improvement in total symptom score, TSS50) and OS. Results showed that 41.9% of patients in the ruxolitinib group, compared to 0.7% in the placebo group, achieved the primary endpoint of spleen response. Additionally, 45.9% of ruxolitinib-treated patients experienced a 50% or more improvement in TSS at 24 weeks, compared to 5.3% in the placebo group. In an updated five-year follow-up of the COMFORT-1 trial, median OS was not reached in the ruxolitinib arm, compared to a median OS of 108 weeks in the placebo arm (censored at crossover) or 200 weeks for all patients (HR, 0.69) [154]. Ruxolitinib was also found to significantly reduce the JAK2 V617F allele burden by 10.9% at week 24 and 21.5% at week 48, compared to placebo. Subgroup analyses showed that the mean reduction in spleen volume and symptom improvement was more pronounced among patients with JAK2 V617F mutations. The activity of ruxolitinib was observed across various myelofibrosis subtypes, including PMF, post-PV MF, and post-ET MF. The most notable side effects were anemia and thrombocytopenia.

The COMFORT-2 trial was an open-label, randomized study comparing ruxolitinib with the BAT [155]. Similar to the COMFORT-1 trial, ruxolitinib resulted in a rapid reduction in splenomegaly and symptom improvement at weeks 24 and 48, with meaningful overall benefits on quality of life. The median duration of response to ruxolitinib was not reached, with 80% of patients still responding at a median follow-up of 12 months.

Although the OS benefit analysis may be limited due to crossover to ruxolitinib and patient withdrawals, the initial COMFORT-2 trial showed a hazard ratio of 0.65 for leukemia-free survival and 0.70 for OS in the ruxolitinib arm. After five years of follow-up, the median OS was not reached in the ruxolitinib arm, compared to 4.1 years in the BAT arm [156]. There was a 33% reduction in the risk of death in ruxolitinib-treated patients (HR = 0.67). Additionally, ruxolitinib suppresses pro-inflammatory cytokine release, including interleukin-6, tumor necrosis factor-alpha, and C-reactive protein, which likely contributed to the improvement in patients’ symptoms. However, there was no change in the bone marrow histomorphology of myelofibrosis associated with ruxolitinib treatment.

**(b). Fedratinib:** Fedratinib, also known as Inrebic, received FDA approval in 2019 for the treatment of intermediate-2 or high-risk primary or secondary myelofibrosis in adults. It is a competitive inhibitor of *JAK2* and also inhibits *FLT3*, *RET*, and *JAK3*. The efficacy and safety of fedratinib were evaluated in the phase III JAKARTA trial, which compared fedratinib to the BAT in JAK inhibitor-naïve patients with intermediate-2 or high-risk primary or secondary myelofibrosis. The results showed significant reductions in spleen volume and total symptom score [157]. The trial was initially placed on hold in 2013 due to concerns about fetal Wernicke encephalopathy. However, additional safety data indicated that no patients experienced Wernicke encephalopathy at the fedratinib dosage of 400 mg/day. On 16 August 2019, the FDA approved fedratinib for treating adult patients with intermediate-2 or high-risk primary or secondary myelofibrosis based on the updated analysis of the JAKARTA trial [158]. The updated trial results demonstrated that fedratinib induced a spleen volume response rate of 47% and a symptom response rate of 40% at week 24, compared to 1% and 9% in the placebo group. While the spleen response was similar to the initial report, the symptom response rate was higher in the updated report (40% vs. 36%) [158].

The efficacy and safety of fedratinib in high-risk MF patients who relapsed or were refractory or intolerant to ruxolitinib were assessed in the FREEDOM2 trial, an open-label, randomized phase 3 study conducted in Europe [159,160]. The results showed that fedratinib was significantly more effective than BAT in reducing spleen size (36% vs. 6%) and providing better symptom relief (34% vs. 17%) in patients previously treated with ruxolitinib. Notably, 21% of patients in the fedratinib group and 4% of patients in the BAT group developed low thiamine levels. As a result, thiamine supplementation is now required for all patients planning to receive fedratinib.

**(c). Pacritinib:** Anemia and thrombocytopenia are common in patients with myelofibrosis. Platelet counts below 50,000/μL prevent the use of ruxolitinib, as dose reductions negatively impact its efficacy in reducing spleen volume [161]. On 28 February 2022, pacritinib, also known as Vonjo, became the first *JAK* inhibitor approved for the treatment of adults with intermediate or high-risk primary or secondary myelofibrosis with a platelet count below 50 × 10^9^/L.

Pacritinib is a multi-kinase inhibitor targeting *JAK2*, *FLT3*, *IRAK1*, and *CSF1R*, but it has minimal activity against *JAK1*. Due to the lack of *JAK1* inhibition, pacritinib causes minimal myelosuppression [162]. The PERSIST-1 trial was a double-blind, randomized, placebo-controlled study that enrolled high-risk myelofibrosis patients, including those with baseline anemia or thrombocytopenia, across 12 countries. It evaluated the efficacy and safety of pacritinib [163]. The results showed that pacritinib induced significant and sustained spleen volume reduction (19% vs. 5%) and symptom relief (36% vs. 14%) compared to the BAT group. The median duration of spleen response was 34.3 weeks, and the mean absolute reduction in spleen volume exceeded 20% at all time points in the pacritinib group. The trial also demonstrated that the reduction in *JAK2* V617F mutation burden correlated directly with symptom improvement in these patients. Major side effects included bleeding and cardiac events. Although the initial study reported that severe bleeding of grade 3–4 occurred in 6% of patients receiving pacritinib, and 12% experienced grade 3–4 cardiac events, a subsequent analysis did not confirm an excess of severe bleeding or cardiac events.

The PERSIST-2 trial was an open-label, randomized controlled trial that enrolled 311 myelofibrosis patients with a prior history of ruxolitinib exposure. It compared the efficacy and safety of pacritinib to the BAT, which included ruxolitinib [164]. The results showed that pacritinib was significantly more effective than BAT in reducing splenomegaly and providing symptom relief in these patients [164]. The pacritinib-induced spleen response was independent of sex, age, *JAK2* V617F mutation status, prior use of *JAK2* inhibitors, and baseline cytopenia. Spleen volume reduction (SVR) is associated with an OS benefit in MF patients with thrombocytopenia [165]. Hematologic and cardiovascular adverse events were similar in both arms.

**(d). Momelotinib:** Momelotinib, also known as Ojjaara, received FDA approval on 15 September 2023 for the treatment of intermediate or high-risk primary or secondary myelofibrosis in adults with anemia. Momelotinib is a selective inhibitor of *JAK1/2* and also targets the ACVR1-mediated expression of hepcidin in the liver. This dual action allows momelotinib to effectively treat myelofibrosis patients with anemia.

The SMIPLIFY-1 phase III trial compared the efficacy and safety of momelotinib versus ruxolitinib in myelofibrosis patients who had not previously been treated with a JAK inhibitor (JAKi) [166]. While only 28% of patients treated with momelotinib achieved a symptom response, which was inferior to the 42% symptom response induced by ruxolitinib, momelotinib significantly reduced transfusion requirements in these patients [166]. However, despite its benefit in treating anemia in MF patients, ruxolitinib appeared to be more effective in reducing fatigue. This suggests that MF-associated fatigue is multifactorial and not solely related to anemia.

The SIMPLIFY-2 trial was an open-label phase III study comparing momelotinib to the BAT, with nearly 90% of patients continuing ruxolitinib, in myelofibrosis patients who had previously been treated with ruxolitinib [167]. The results showed no significant difference in spleen response (SVR35) between the two groups (7% vs. 6%). However, there was a significant total symptom response (TSS50) in the momelotinib group (26% vs. 6%). Therefore, momelotinib provided greater symptom relief for patients who were previously treated with ruxolitinib, despite no difference in spleen response. It is important to note that patients in the SIMPLIFY-2 trial were not truly ruxolitinib-resistant but rather suboptimal responders or intolerant of ruxolitinib.

Among myelofibrosis patients with symptomatic anemia, momelotinib stands out as the only JAK2 inhibitor that increases erythropoiesis by inhibiting ACVR1-mediated hepcidin expression. In the phase III MOMENTUM trial, momelotinib demonstrated superiority over danazol in terms of total symptom score (TSS) response, transfusion independence (TI) rate, and splenic response rate (SRR) [168]. A long-term pooled data analysis from the MOMENTUM, SIMPLIFY-1, and SIMPLIFY-2 trials showed a survival benefit in patients receiving momelotinib, with survival rates of 76.5%, 59.6%, and 51.1% in years 2, 4, and 6, respectively. The median OS has not yet been reached [169].

#### 4.2.5. Emerging Therapies Beyond Frontline JAK Inhibitors

Over several years of follow-up in clinical trials, it has been observed that 50% of myelofibrosis patients discontinue ruxolitinib after three years. The survival rate for these patients after JAK inhibitors is poor, averaging 11 to 16 months. The JAK-refractory MF is of higher risk of disease progression and represents a significant unmet medical need.

(a). Navtemadlin: *MDM2* inhibitor

In the context of MF, MDM2 (mouse double minute 2 homolog) is a protein that negatively regulates the tumor suppressor protein p53. The overexpression of *MDM2* has been documented in CD34+ MF cells, leading to the attenuation of p53 activity and proliferation of malignant cells. Navtemadlin is a small-molecule inhibitor targeting *MDM2*. By inhibiting *MDM2*, navtemadlin restores p53 activity, leading to apoptosis (programmed cell death) in cancer cells. The efficacy of navtemadlin versus the BAT was evaluated in the randomized, global phase 3 BOREAS study, which enrolled *TP53*-wild-type patients with myelofibrosis who relapsed or are refractory to JAK inhibitor treatment. The results of the BOREAS trial, presented at ASCO 2024, demonstrated that navtemadlin significantly reduced spleen volume and improved symptom scores compared with the BAT [170]. Very importantly, navtemadlin also showed evidence of disease modification, with nearly half of the patients (48%) experiencing a reduction in bone marrow fibrosis and sustained diver gene VAF reduction [170].

(b). Selinexor: XPO1 inhibitor

Combining selinexor and ruxolitinib has shown promising results for the treatment of myelofibrosis. Selinexor is an oral selective inhibitor of nuclear export protein 1 (XPO1). Together with ruxolitinib, these drugs target different pathways involved in the disease process. A phase 2 study evaluated the efficacy and safety of this combination in MF patients who were either resistant to or had a suboptimal response to ruxolitinib. The interim analysis showed that nearly 71% of patients experienced a reduction in spleen volume, and 38% achieved a spleen response [171]. Additionally, there were improvements in symptom scores and anemia responses [172]. The combination of selinexor and ruxolitinib versus ruxolitinib alone is now also being studied in the SENTRY (XPOT-MF-034) phase trial for JAK-naïve myelofibrosis.

(c). Pelabresib: BET inhibitor

Pelabresib is an investigational oral small-molecule drug designed to inhibit bromodomain and extra-terminal domain (BET)-mediated gene transcription. BET proteins regulate the transcription of a set of genes that lead to various oncogenic abnormal signals. BET inhibition has the potential to modify multiple critical components of myelofibrosis (MF) pathobiology, including megakaryocyte differentiation and proliferation. The phase III MANIFEST-2 trial evaluated the efficacy and safety of pelabresib combined with ruxolitinib versus placebo combined with ruxolitinib in patients with JAK inhibitor treatment-naive MF [173]. Initial results showed that pelabresib, when combined with ruxolitinib, significantly reduced spleen volume and improved anemia in patients with MF. Updated results from the MANIFEST-2 trial, presented at ASH 2024, demonstrated a continued deep and sustained spleen response (SVR35), with a higher proportion of patients maintaining the response, a higher rate of hemoglobin response, fewer patients requiring blood transfusions, and an increased rate of TSS50 response at 48 weeks in patients receiving both pelabresib and ruxolitinib [174]. Remarkably, there was a continued improvement in bone marrow fibrosis and sustained reductions in levels of disease-relevant pro-inflammatory cytokines, with a trend toward a greater reduction in mutant clone burden, suggesting a potential disease-modifying benefit of pelabresib when combined with ruxolitinib [174].

(d). Managing Anemia in MF

Anemia is observed in 35% to 54% of myelofibrosis patients at diagnosis and is linked to a decreased quality of life, shorter survival, and an increased risk of leukemic transformation [175]. The incidence of anemia worsens over time, with up to 46% of patients becoming dependent on red blood cell (RBC) transfusions within a year of diagnosis [175]. Most JAK inhibitors exacerbate anemia during treatment, presenting a significant challenge in managing myelofibrosis patients. Traditional treatments for MF-associated anemia include red blood cell transfusions, erythropoiesis-stimulating agents (ESAs), androgens like danazol, and immunomodulatory agents such as lenalidomide and thalidomide. The recently approved luspatercept, which targets the bone morphogenetic protein-SMAD signaling pathway, and momelotinib, a newer JAK1/JAK2 inhibitor with anemia-related efficacy, have shown benefits in managing anemia in myelofibrosis.

Anemia in myelofibrosis is multifactorial, with contributing factors including elevated hepcidin levels due to inflammation and cytokine release, decreased iron availability for erythropoiesis, and the use of JAK inhibitors. Hepcidin levels in MF are significantly elevated—up to 12 times higher—and are associated with the severity of anemia and the burden of transfusions [176]. DISC-0974 is a groundbreaking monoclonal antibody targeting hemojuvelin (HJV), a co-receptor in the bone morphogenetic protein (BMP)-signaling pathway that drives hepcidin expression. By inhibiting hepcidin, DISC-0974 enhances iron absorption from the gut and promotes the release of iron from macrophages, ultimately increasing iron availability for maintaining homeostasis. Initial results from the phase 1b study for DISC-0974, presented at ASCO 2024, showed promising efficacy in improving anemia in patients with myelofibrosis [177]. During the study period, 68% of non-transfusion-dependent MF patients achieved a hemoglobin (Hb) increase of more than 1.5 g/dL, and 50% maintained a sustained Hb response for over 12 weeks [177]. Additionally, a significant number of transfusion-dependent patients experienced a major reduction in transfusion requirements or achieved transfusion independence [177]. Furthermore, more than 50% of patients receiving concomitant JAK inhibitors also achieved a major hematologic response.

## 5. Conclusions

In modern medicine, MPNs are recognized as a group of rare blood cancers characterized by the uncontrolled production of red blood cells, white blood cells, or platelets within the bone marrow. Recent advancements in genetic sequencing and artificial intelligence (AI) are revolutionizing cancer research, including understanding the molecular aspect and treatment of MPNs. Genomic and transcriptome next-generation sequencing (NGS) has unveiled a highly intricate and complex mutational landscape for MPNs, both at diagnosis and following therapy. While well-known driver mutations such as *JAK2*, *CALR*, and *MPL* play a central role in MPN development, emerging research indicates the significance of passenger mutations or co-mutations in shaping the evolution of MPN clones. The aberrant signaling activation derived from both driver and passenger mutations contributes to chronic inflammation, dysregulated cytokine release, a hypercoagulable state, and abnormal iron metabolism. These interconnected factors define the complex pathophysiology of MPN influencing clinical manifestation and complications, including thrombosis, vascular complications, and leukemic transformation.

Due to their complex biology, MPNs pose significant challenges in treatment. The primary objectives are to achieve remission, improve quality of life and survival, and minimize the risk of blood clots and complications. Advancements in our understanding of the molecular mechanisms driving MPNs have identified numerous therapeutic targets, leading to the development of novel treatment strategies. The introduction of *JAK* inhibitors has revolutionized the management of symptomatic myelofibrosis. Additionally, emerging therapies targeting *MDM2*, *BET*, *XPO1*, and *BCL* are undergoing intensive studies, either alone or in combination with *JAK* inhibitors, to improve therapeutic outcomes, particularly for patients who have failed *JAK* inhibitors. One of the greatest challenges in myelofibrosis is anemia, which arises from both the disease itself and treatment-related side effects. This condition significantly impacts quality of life and is associated with poor survival. New approaches, such as small interfering RNA (siRNA)-based drugs, aim to modulate iron metabolism and enhance erythropoiesis, offering hope for improved anemia management in the future. Lastly, CML is no longer regarded as a single mutation-driven disease. The complexity of somatic mutations and their evolving dynamics—both at diagnosis and throughout TKI therapy—has revealed a more intricate pathophysiology, potentially shaping future therapeutic strategies and personalized treatment.

## Figures and Tables

**Table 1:** Diagnosis of high-risk CP-CML or AP-CML.

WHO 5th Edition: High Risk CP-CML		2022 ICC: AP-CML
At diagnosis	High ELTS score	
	10–19% blasts in the PB and/or BM	10–19% blasts in the PB and/or BM
	≥20% basophils in the PB	≥20% basophils in the PB
	Additional clonal chromosomal abnormalities in Ph+ cells *	Additional clonal chromosomal abnormalities in Ph+ cells at diagnosis *
	Clusters of small megakaryocytes associated with significant reticulin and/or collagen fibrosis in biopsy	
**Emerging on treatment**	Resistance to TKI, including loss of prior responses, emergence of ACA and *BCR::ABL1* kinase domain mutations	

* Additional chromosomal abnormality: Second Ph, trisomy 8, isochromosome 17q, trisomy 19, complex karyotype, abnormalities of 3q26.2, monosomy 7, 11q23 rearrangements, or trisomy 21.

**Table 2:** Revised classification and diagnostic criteria for *BCR::ABL* negative MPN in 2022 [2].

Disease	Diagnostic Criteria
PV	**3 major or the first 2 major + 1 minor** **3 Major criteria** PB: ↑ Hb (>16.5 g/dL in men; >16.0 g/dL in women) or ↑ Hct (>49% in men; >48% in women) BM: age-adjusted hypercellularity with panmyelosis with pleomorphic mature megakaryocytes (differences in size) Presence of *JAK2* V617F or *JAK2* exon 12 mutation **1 minor criterion** Subnormal serum EPO level
Post-PV MF	**All required criteria + at least 2 additional criteria** **Required criteria** Documentation of a previous Dx of WHO-defined PV Bone marrow fibrosis of grade 2–3 on a 0–3 scale **Additional criteria** (2 are required) Anemia or sustained loss of requirement of either phlebotomy or cytoreductive treatment for erythrocytosis Leukoerythroblastosis Increasing splenomegaly in palpable splenomegaly of >5 cm from baseline or the development of a newly palpable splenomegaly Development of any 2 (or all 3) of the following constitutional symptoms: >10% weight loss in 6 months, night sweats, unexplained fever (>37.5 °C)
ET	**All major criteria or the first 3 major + minor criterion** **Major criteria** PB: Platelet count ≥ 450 × 10^9^/L BM: showing proliferation mainly of the megakaryocytic lineage, with increased numbers of enlarged, mature megakaryocytes with hyperlobated nuclei; no significant increase or left shift in neutrophil granulopoiesis or erythropoiesis; very rarely a minor (grade 1) increase in reticulin fibers WHO criteria for *BCR-ABL1*-positive CML, PV, PMF, or other myeloid neoplasms are not met **Minor criterion:** Presence of a clonal marker or absence of evidence of reactive thrombocytosis
Post ET MF	**All required criteria + at least 2 additional criteria** **Required criteria** Documentation of a previous Dx of WHO-defined ET Bone marrow fibrosis of grade 2–3 on a 0–3 scale **Additional criteria (2 are required)** Anemia and a >2 g/dL decrease from baseline Hb Leukoerythroblastosis Increasing splenomegaly: ↑ in palpable splenomegaly of >5 cm from baseline or the development of a newly palpable splenomegaly ↑ LDH (above reference range) Development of any 2 (or all 3) of the following constitutional symptoms: >10% weight loss in 6 months, night sweats, unexplained fever (>37.5 °C)
Prefibrotic PMF	**All 3 major criteria + at least 1 minor** **Major criteria** Megakaryocytic proliferation and atypia, without reticulin fibrosis grade > 1, accompanied by ↑ age-adjusted BM cellularity, granulocytic proliferation, and (often) ↓ erythropoiesis WHO criteria for BCR-ABL1-positive CML, PV, ET, MDS, or other myeloid neoplasms are not met *JAK2*, *CALR*, or *MPL* mutation; or presence of another clonal marker (e.g., *ASXL1*, *EZH2*, *TET2*, *IDH1*, *IDH2*, *SRSF2*, and *SF3B1* mutations); or absence of minor reactive bone marrow reticulin fibrosis * Minor criteria (at least 1, confirmed in 2 consecutive determinations) Anemia not attributed to a comorbid condition Leukocytosis ≥ 11 × 10^9^/L Palpable splenomegaly ↑ LDH (above reference range)
Fibrotic PMF	**All 3 major criteria + at least 1 minor** **Major criteria** Megakaryocytic proliferation and atypia, accompanied by reticulin and/or collagen fibrosis grades 2 or 3 WHO criteria for *BCR-ABL1*-positive CML, PV, ET, MDS, or other myeloid neoplasms * are not met *JAK2, CALR*, or *MPL* mutation; **or** presence of another clonal marker (e.g., *ASXL1, EZH2, TET2, IDH1, IDH2, SRSF2*, and *SF3B1* mutations); **or** absence of reactive fibrosis **Minor criteria (at least 1, confirmed in 2 consecutive determinations)** Anemia not attributed to a comorbid condition Leukocytosis ≥ 11 × 10^9^/L Palpable splenomegaly ↑ LDH (above reference range) Leukoerythroblastosis

**Table 3:** Mutation-Enhanced International Prognostic Scoring System (MIPSS70/v2) and the Genetically Inspired Prognostic Scoring System (GIPSS).

	Clinical	Karyotype	Driver Mutation	HMR Mutations
GIPSS	Not included	Very high risk (2 points) Unfavorable (1 point)	Absence of type 1-like CALR (1 point)	ASXL mutation (1 point) SRSFZ mutation (1 point) UZAPI Q157 mutation (1 paint)
MIPSS70+v2.0	Severe anemia: Hb < 9 (M) or 8 (W) g/dL (2 points) Moderate anemia: Hb 9 to 10.9 (M) or 8 to 9.9 (W) g/dL (1 point) Circulating blasts = 2% (1 point) Constitutional symptoms (2 points)	Very high risk (4 points) Unfavorable (3 points)	Absence of type 1-like CALR (2 point)	>1 HMR mutations (3 points) 1 HMR mutation (2 point)

**Table 4:** Prognostic impact of mutations in Ph-negative MPNs [72].

Driver Mutations	PV	ET	PMF
*JAK2*	Increased risk of thrombosis and disease progression	Increased thrombosis risk	An intermediate prognosis and an elevated risk of thrombosis compared to patients with *CALR* mutation
*CALR* (frameshift in exon 9)		Lower risk of thrombosis compared to JAK2 mutated	*CALR* type 1 are associated with improved survival compared to *JAK2 V617F* and triple-negative cases, with better outcomes following SCT. CALR type 2 mutations lower OS compared to CALR type 1 [65]
*MPL* (exon10)			Intermediate prognosis and increased thrombosis risk compared to *CALR* mutation patients
